# Effects of food price on nutrition outcomes among women in Nigeria

**DOI:** 10.1002/fsn3.3737

**Published:** 2023-10-03

**Authors:** Deborah Tosin Fajobi, Joshua Olusegun Ajetomobi, Mufutau Oyedapo Raufu, Moses Oluwatobi Fajobi, Prabhu Paramasivam

**Affiliations:** ^1^ Department of Agricultural Economics, Open and Distance Learning Centre Ladoke Akintola University of Technology Ogbomoso Nigeria; ^2^ Department of Agricultural Economics, Faculty of Agricultural Sciences Ladoke Akintola University of Technology Ogbomoso Nigeria; ^3^ Department of Mechanical Engineering University of Ilorin Ilorin Nigeria; ^4^ Open and Distance Learning Centre Ladoke Akintola University of Technology Ogbomoso Nigeria; ^5^ Department of Mechanical Engineering, College of Engineering and Technology Mattu University Mettu Ethiopia

**Keywords:** food price, nutrition and R packages, obesity, overweight, underweight, women

## Abstract

Nutrition outcomes (undernutrition, overweight, and obesity) among women are growing concerns across the globe. Currently, the rate of undernutrition and overweight among women in Nigeria is ranked among the highest in Africa. A major contributory factor reported is unstable food prices in the country. This study, therefore, examined the effects of food prices on nutrition outcomes among women in Nigeria. Secondary datasets retrieved from two different sources were used for this study. Cross‐sectional data on weight and height for women were obtained from Nigeria Health Demographic Survey (NHDS). Data on monthly prices of the selected food items were obtained from the Nigeria Bureau of Statistics (NBS). The data were categorized into energy dense (yam tuber, *garri*, rice, and maize) and nutrient dense (egg, beef, and chicken). Multinomial logit regression was used to estimate the relationship between the prices of energy and nutrient‐dense food prices concerning respondents' personal and environmental characteristics such as age, wealth status, and region; as well as the three nutrition outcomes for women (undernutrition, overnutrition, and obesity). This study revealed that the prevalence of overweight and obesity among women was 19.9% and 10.3%, respectively. Nutrition outcomes (obesity and overweight) were positively correlated with the price of energy‐dense food with 0.2% and 0.3%, respectively. Nutrient‐dense food price is negatively correlated with undernutrition with a probability of 0.1%. The study recommends that food policy instruments such as food prices and subsidies can be introduced to favor the consumption of healthier food to stem the prevalence of overweight and obesity in Nigeria.

## Graphical abstract

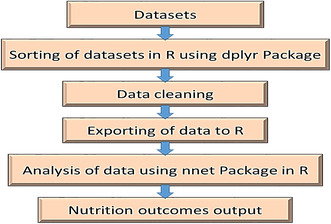

## 1 INTRODUCTION

In emerging countries, adult undernutrition, overweight, and obesity are on the rise. These challenges are spreading to poorer countries due to excessive consumption of energy‐dense meals and sedentary lifestyles (Amin et al., 2015; Neupane et al., 2015; Onyeaka et al., 2023). Fifteen years before the year 2018, Nigeria Health Demographic Survey (NDHS, 2018) reported that undernutrition, overweight, and obesity have been on the rise in Nigeria and other emerging nations. Nearly 2 billion overweight or obese people are reported to be living in developing countries (Shekar & Popkin, 2020). High overweight and obesity rates are connected with a high incidence of noncommunicable diseases (NCDs), which are now among the major causes of death worldwide (Kavle et al., 2022; Shekar & Popkin, 2020). Food prices influence consumption habits but high food prices can affect nutrition and health (Green et al., 2013). Increment in food prices triggers consumers to make unintended adjustments in terms of nutritional content. Since Nigeria is a major importer of food, any international food market crisis will adversely impact Nigerians, consequently, increasing the food price and the price of the food items (Green et al., 2013). Therefore, it will amount to a deterioration in food intake of calories and protein as people engage in unhealthy food consumption patterns (Amin et al., 2015). The high cost of nutrient‐dense foods may affect nutrition outcomes. A rise in relative prices might reduce the intake of nutritious foods, depending on the country and type of food (Miller et al., 2016). The cost of food is an important driver of food choice, and most evidence suggests that healthier diets are costlier than less healthy diets (Jody et al., 2022).

The cost of nutrient‐dense foods correlates with overall diet quality and nutrition implications like undernutrition, overweight, and obesity (Headey, 2017). It is necessary to note that those with less financial resources are more likely to be negatively impacted by the increased cost of wholesome foods. They are more price sensitive, and as a result, they are more prone to go for diets that are less expensive but have a higher concentration of energy (Darmon et al., 2014; Miller et al., 2016). Even though the price is not the sole factor that determines diets and nutrition outcomes, the cost of good and unhealthy meals is an essential one, and it is likely to be more binding for those who are poor than for those who are not poor. Previous research has been carried out to investigate the effects of food prices on various nutrition outcomes. The details of the peculiar outcomes, price measures, and sources of data used in the previous studies are shown in Table 1. It was evident that BMI was the most commonly used outcome indicator while the prominent price measures were fruits, vegetables, eggs, milk, fast food, sugar, potatoes, cereals, and maize. The present study partitioned the BMI into undernutrition, overnutrition, and obesity to better understand the effects of food prices on nutrition outcomes. This paper explores important measures of food price (energy‐dense and nutrient‐dense food) which has received less attention in the literature.

**TABLE 1:** Nutrition outcomes/BMI reported by different researchers.

Au authors	Topic	Outcomes considered	Price measure	Source of dataset	Findings
Chou et al. (2004)	An economic analysis of adult obesity: results from the Behavioral Risk Factor Surveillance System	BMI and Obesity	Restaurant prices, fast food restaurant prices, and ACCRA	Behavior Risk Factor Surveillance System 1984–1999	All the prices have negative effects on BMI and obesity
Asfaw (2006)	The role of food price policy in determining the prevalence of obesity: evidence from Egypt	BMI	Prices of sugar, oil, rice, fruits, vegetables, egg, milk, and beef	Egyptian Rural Household Survey 1997	Prices of sugar, oil, egg, and milk negatively influence BMI. Price of fruits is positive
Powell et al. (2006)	Access to fast food and food prices: relationship with fruit and vegetable consumption and overweight among adolescents	BMI	Fruit, vegetables, and fast food from ACCRA	Monitoring the Future Survey 1997–2003	Fruit and vegetable price elasticities are positive while fast food piece elasticities are negative
Beydoun et al. (2008)	The association of fast food, fruit, and vegetable prices with dietary intakes among US adults: Is there modification by family income?	BMI and obesity	Fruit, vegetables, and fast food from ACCRA	Continuing survey of food intakes by individuals 1994–1996	No significant relationship between fast food and BMI and obesity. Fruit and vegetable prices have no effects on BMI and obesity
Miljkovic et al. (2008)	Economic factors affecting the increase in obesity in the United States: Differential responses to price	Overweight and obesity	Prices of sugar, potatoes, and whole milk from USDA National Agricultural Statistics	Behavior Risk Factor Surveillance System 1991, 1997, and 2002	The price of potatoes positively affects the probability of obesity while the price of whole milk and sugar has negative effects on the probability of obesity
Darmon et al. (2014)	Food price policies improve diet quality while increasing socioeconomic inequalities in nutrition	NA	A fruit and vegetable price subsidy and an unhealthy product tax named nutrient profile condition	The nutrient profiling system called SAIN and LIM was used	The findings imply that food price policies may increase socioeconomic nutrition inequalities while improving diet quality
Ajetomobi et al. (2019)	Effects of food price policies on prevalence of overweight and obesity in the Kingdom of Eswatini	BMI and Obesity	Egg, milk, vegetable, fruit, maize, beef, goat, and oil from restaurants and stores prices	Swaziland Demographic and Health Survey 2006–2007	BMI is negatively correlated with the price of cooking oil relative to the price of maize but positively correlated with the price of vegetables and fruit. Age, household size, urbanization, and level of wealth have positive and significant effects on BMI
Brenton and Nyawo (2019)	Food prices, access to markets, and child undernutrition in Ethiopia	Child undernutrition	Market prices for cereals, teff, wheat, and maize	Livings Standards Measurement Survey	For children between the ages of 6 months and 5 years, improved rates of child stunting are positively correlated with rising crop prices
Authors	Effects of food price on nutrition outcomes among women in Nigeria	Undernutrition, overnutrition, and obesity	Prices of energy dense and nutrient dense	Nigeria Demographic Health Survey 2018	The price of energy dense is positively correlated with obesity and overnutrition while nutrient dense is negatively correlated with undernutrition

Abbreviation: NA, not applicable.

This information is essential for developing policies and initiatives to improve the living standards of Nigerian households.

According to statistics, food prices in Nigeria have risen over time. The consumer food price index rose from 71.9 points in 2007 to 109.9 points in 2010 (CBN, 2012). In the year 2012, it reached 134.9 points, and in 2015, 186.2 points (CBN, 2016). It reached 261 points, then 278.2 points in June 2018. August 2019 was 323.9 points and December 2019 was 339.9 points, while it reached 447.2 points in July 2021 (Nigeria Bureau of Statistics, 2021). Based on some selected food commodities, the National Bureau of Statistics released food price watch data in June 2021, the average price per 1 kg of imported rice increased by 39.6%, Also, the average price of one egg (medium size) increased by 34.6%, and the average price per 1 kg of tomato rose by 13.0%. One kg of yam tuber climbed 52.7% (Nigeria Bureau of Statistics, 2021).

*Garri* rose by 65.8%, beans rose by 42.7%, while beef prices surged up to 29.9%, fish prices rose by 60.2%, and local rice prices rose by 37.4% (Nigeria Bureau of Statistics, 2021). Poor and vulnerable households spend up to 80% of their incomes on food (Obayelu, 2010). When price or income shocks hit, households slash their food budget first (Ayinde et al., 2012; Capuno et al., 2013). Nutritional intakes decline in quantity and quality, causing food insecurity, malnutrition, poverty, and other problems (Gebremichael & Belachew Lema, 2023). Also, many households in the country eat fewer and lower‐quality foods (Agada & Igbokwe, 2015; Ajani, 2010; Akerele, 2015). Several country case studies revealed that the prevalence of obesity, overweight, and undernutrition can be connected to improper eating habits, food prices, a lack of health consciousness, availability to fast food outlets and supermarkets, and government food subsidy programs on relative price policy (Asfaw, 2006; Galal, 2002; Perrin et al., 2018; Powell et al., 2009; Viola et al., 2013). Despite the prevalence of the problems of nutrition outcomes (underweight, overweight, and obesity) in the country, nationwide research studies are yet to be carried out on the determinants. Much less is known about the relationship between women's nutrition outcomes and food prices in Nigeria. Hence, the present study used multinomial logit regression using the conventional software package (R‐Studio, version 2021) to determine the relationship between them.

## 2 LITERATURE REVIEW

Until this moment, a few empirical research carried out to explore the association between food prices and body mass index (BMI) is not popular. For instance, Lakdawalla and Philipson (2002) assessed the contribution that food costs make to the rising prevalence of obesity in the United States by utilizing regional differences in food taxes. The authors found that a reduction in supply price resulted in a 0.72‐unit increase in BMI between 1981 and 1994. Sturm and Datar (2005) assessed the relationship between changes in body mass index (BMI), food prices, and food outlet density, among elementary school kids in the United States. The harvested data were analyzed for mean least‐squares regression, 85th percentile median, and quantile regression (Sturm & Datar, 2005). The study established that there is a proportional relationship between the price of fruits and vegetables and the amount of weight gain experienced by kindergarten and third‐grade children in America, such that observed reduction in one indicated a corresponding decrease in the other and vice versa. Asfaw (2006) examined the impact of the Egyptian food subsidy program on mothers' weight. The author evaluated the nexus between the costs of nine different food groups (prices of sugar, oil, rice, fruits, vegetables, egg, milk, and beef) and the average BMI of Egyptian women and used a home survey with a single cross‐section alongside with seven different iterations of monitoring the future surveys (1997–2002). The author established that there are significant negative consequences for products that are high in energy density and favorable effects for products that are lower in energy density. Powell and Chaloupka (2009) investigated the relationship among teenage consumption of fruits and vegetables, body mass index (BMI), and likelihood of being overweight and the relationship between food prices and restaurant outlet density. They found that the cost of fast‐food meals significantly influenced adolescents' body weight and eating habits. A 10% increase in the price of fast food resulted in a 3.0% increment in the likelihood of frequently consuming fruits and vegetables, a decrease of 0.4% in BMI, and an increase of 5.9% in the likelihood of being overweight.

Ivanic and Martin (2008) conducted research on the welfare and poverty impacts of changes in the world prices of key staple food commodities in nine nations (Bolivia, Cambodia, Madagascar, Malawi, Nicaragua, Pakistan, Peru, Vietnam, and Zambia) with low incomes and concluded that an increase in the cost of staple foods led to a rise in poverty in the majority but not all of those countries. Vellakkal et al. (2015) investigated the connection between rising food prices in the 2000s and the prevalence of childhood malnutrition in India. The authors first examined the connection between food consumption and food price fluctuations in child nutrition. The results of the study indicated that a rise in food prices is connected to a higher risk of childhood malnutrition. Arndt et al. (2016) investigated the connection between variations in food prices and the nutritional standing of children in Mozambique by using household survey data and food price inflation rates as their primary data sources. The research indicates, via the use of a technique called propensity score matching, that nutritional measures (wasting and stunting) significantly improved when the inflation rate for food products was low. However, their analysis focused on general price inflation and covers changes between quarters within 1 year so does not allow for longer‐term impacts.

Woldemichael et al. (2017) investigated the impact of teff, maize, and wheat price inflation on a child's health at various stages of development in the months before and after birth. The authors used information from numerous rounds of the Demographic and Health Surveys as well as separate information on pricing at the local level to develop a U‐shaped link between food price inflation and a child's height for age (stunting). Compared to wheat and maize, teff had more significant impact. In terms of wasting, food price inflation had little impact on a child's development (weight for height). Nutrition and evaluation of nutritional status among children under the age of 5 have received a lot of attention in developing nations like Nigeria (Adeoya et al., 2023; Nkonde et al., 2021), but nutrition among women has received less consideration and examination. Furthermore, the awareness of food price and its impacts on body mass index among women is low and there is the misperception that food price does not affect nutrition outcomes. Against these backdrops, this study looks into how women's nutrition and their personal environment are affected by food prices in Nigeria.

## 3 METHODS

**FIGURE 1:**
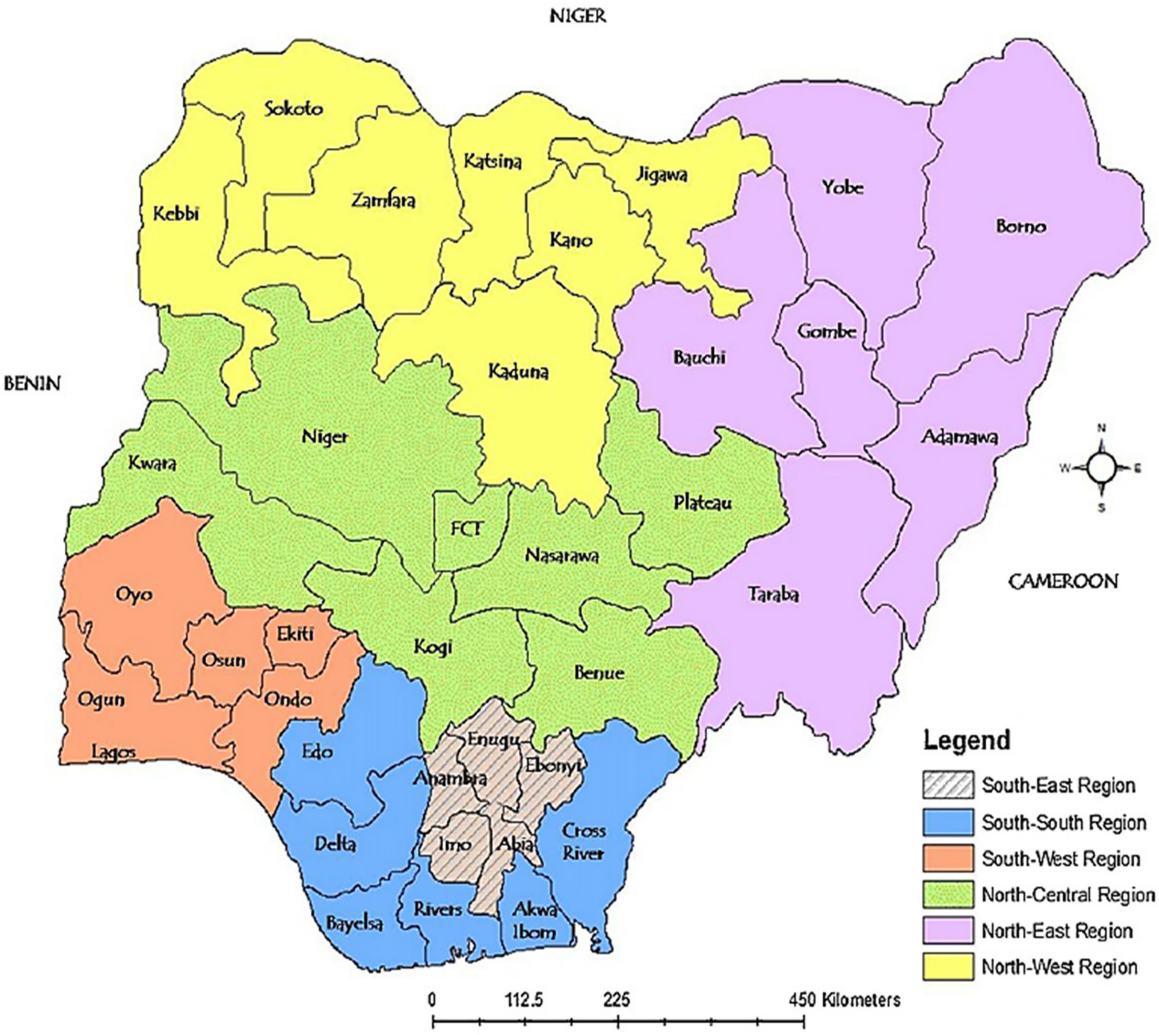
Map of Nigeria showing the six geopolitical zones and the 36 states. Source: Wikipedia <https://en.wikipedia.org/wiki/Nigeria>.

This study was carried out in Nigeria. The Federal Republic of Nigeria is located in the tropical zone of West Africa between latitudes 4°N and 14°N and longitudes 2°2′ E and 14°30′ E with a total land area of 923,770 km^2^ (Wikipedia). Nigeria is a nation with six geographical zones namely North‐West, South‐West, North‐East, South‐East, North‐Central, and South‐South (Figure 1). Cereal crops (rice, millets, sorghum, etc.) which are the most energy‐dense food were cultivated and prominent in the northern part while yam, cassava, and maize were major staple foods cultivated and prominent in the eastern and western parts of the country (Nigeria Bureau of Statistics, 2021).

### 3.1 Sources of data and sampling

This cross‐sectional design study involved the analysis of secondary data collected on women during the 2018 Nigeria Demographic and Health Survey (NDHS). The NDHS survey was carried out to provide reliable information about men's, women's, and children's health and family planning services in the urban and rural areas, across the country's six geographical zones, and in each of the 36 states and the Federal Capital Territory. A multistage random sampling strategy was used to choose the eligible respondents, who were women of reproductive age (15–49 years) for this nationally representative sample survey that included the whole population living in noninstitutional dwelling units in Nigeria. The sample size for the survey is 4907 women in the age range of 15–49 years selected among 7871 households, making it a nationally representative study. The survey provides information on health and demographic indicators at the national level, for urban–rural areas, and for the six geopolitical zones in Nigeria (namely North‐Central, North‐East, North‐West, South‐West, South‐East, and South‐South). The NDHS dataset is used not just for height and weight indicators but also for individual‐level controls.

In the study, respondents who were pregnant were not captured in the data used for analysis to avoid unnecessary weight shocks. The sample was weighted to ensure that it is nationally represented. There are many missing data in the Nigeria Demographic Health Survey, and these were handled with the use of a mice package in R‐Studio. Data on prices of food items for this study were obtained from the National Bureau of Statistics (NBS) which was covered in 2018 for selected food prices and it was collected on monthly basis and locations corresponding to NDHS data but no missing data. The reliability of the data was checked by comparing the prices with price information from small shops and supermarkets located in various regions. The prices are average state market prices over 12 months' period for each food item.

### 3.2 Classification of foods

Food items were categorized into energy‐dense (yam tuber, *garri*, maize, and rice) and nutrient‐dense food (chicken, egg, and beef) using the Food Standards Agency in Nigeria (2002) which provides a categorical definition of a food's healthiness based on energy and protein.

### 3.3 Statistical analysis

The analysis of the data involves the use of the following R packages: nnet and Hmisc version 4.0.4. Percentage distribution means and standard deviations (SD) were calculated for each item used. Multinomial regression analysis was used to determine the effect of food price on BMI and personal and environmental characteristics (place of residence, wealth status, age, region, and educational level) based on the variable defined in Table 2. The level of significance was set to *p* < .01, .05, and .1, respectively.

**TABLE 2:** Definition of variables.

Variable	Definition
Body Mass Index (BMI_w1_)	Weight (kg/m^2^): BMI ≤ 18.5 kg/m^2^ indicates underweight, 19 ≥ BMI ≤ 24.9 kg/m^2^ indicates normal weight, 25 ≥ BMI ≤ 29.9 kg/m^2^ indicates overweight, and BMI > 30.0 kg/m^2^ indicates obesity
Personal characteristics (𝑋_w1_)	
Age	Years
Education	No formal education = 0, elementary education =1, secondary education = 2, tertiary education = 3
Household size	Number
Wealth status	Rich = 1, Poor = 0
Urbanization	If area is urban, 1. If area is rural, 0
Price indicators (𝑃_𝑖𝑗_)	
Price of energy dense (rice, maize, yam tuber, and maize per kg)	Local currency ( **N** )
Price of nutrient dense (egg, beef, and chicken per kg)	Local currency ( **N** )

### 3.4 Outcome variable includes obesity, overweight, and undernutrition

The body mass index (BMI) is the most widely used of all numerous internationally recognized anthropometric indexes (Fatima et al., 2023). All the nutrition outcomes among adults were assessed by using body mass index (BMI; kg/m^2^) ranges shown in Equation (1) (Fatima et al., 2023).

BMI is defined as the person's weight in kilograms divided by the square of the height in meters. When BMI < 18.5 kg/m^2^ indicates underweight, 19 ≥ BMI ≤ 24.9 kg/m^2^ indicates normal weight, 25 ≥ BMI ≤ 29.9 kg/m^2^ indicates overweight, and BMI > 30.0 kg/m^2^ indicates obesity (WHO, 2019). BMI used in the multinomial regression analysis was coded as 1 if the respondent has any of the nutrition outcomes (obesity, overweight, and underweight) and coded as 0 if otherwise.

(1) $$\mathrm{BMI}=\frac{\text{Body weight}\;\left(\mathrm{kg}\right)}{\text{Square of height}\;\left(m^{2}\right)\;}$$

### 3.5 Empirical model specification

Theoretically, food prices and access to food outlets may influence BMI through the consumption of food items (Ajetomobi et al., 2019; Orazio et al., 2009). The basic model for this study, therefore, is shown in Equations 2 and 3.

### 3.6 Multinomial logit equation model for energy‐dense food price for women

(2) $${\mathrm{BMI}}_{W1}=\beta_{0}+\beta_{1}X_{1}+\beta_{2}X_{2}+\beta_{3}X_{3}+\beta_{4}X_{4}+\beta_{5}X_{5}+\beta_{6}X_{6}$$

where:

BMI_w1_ = obesity, overweight, and underweight; $\beta_{0}$ = constant of parameters to be estimated; $\beta_{1}\ldots \beta_{8}$ = the vector of the parameters to be estimated; *X*_1_ = place of residence; *X*_2_ = wealth status; *X*_3_ = age; *X*_4_ = region; *X*_5_ = education; *X*_6_ = energy‐dense food price.

### 3.7 Multinomial logit equation model for nutrient‐dense food price for women

(3) $${\mathrm{BMI}}_{W2}=\beta_{0}+\beta_{1}X_{1}+\beta_{2}X_{2}+\beta_{3}X_{3}+\beta_{4}X_{4}+\beta_{5}X_{5}+\beta_{6}X_{6}$$

where:

BMI_w2_ = obesity, overweight, and underweight; $\beta_{0}$ = constant of parameters to be estimated;$\;\beta_{1}\ldots \beta_{8}$ = the vector of the parameters to be estimated; *X*_1_ = place of residence; *X*_2_ = wealth status; *X*_3_ = age; *X*_4_ = region; *X*_5_ = education; *X*_6_ = nutrient‐dense food price.

**FIGURE 2:**
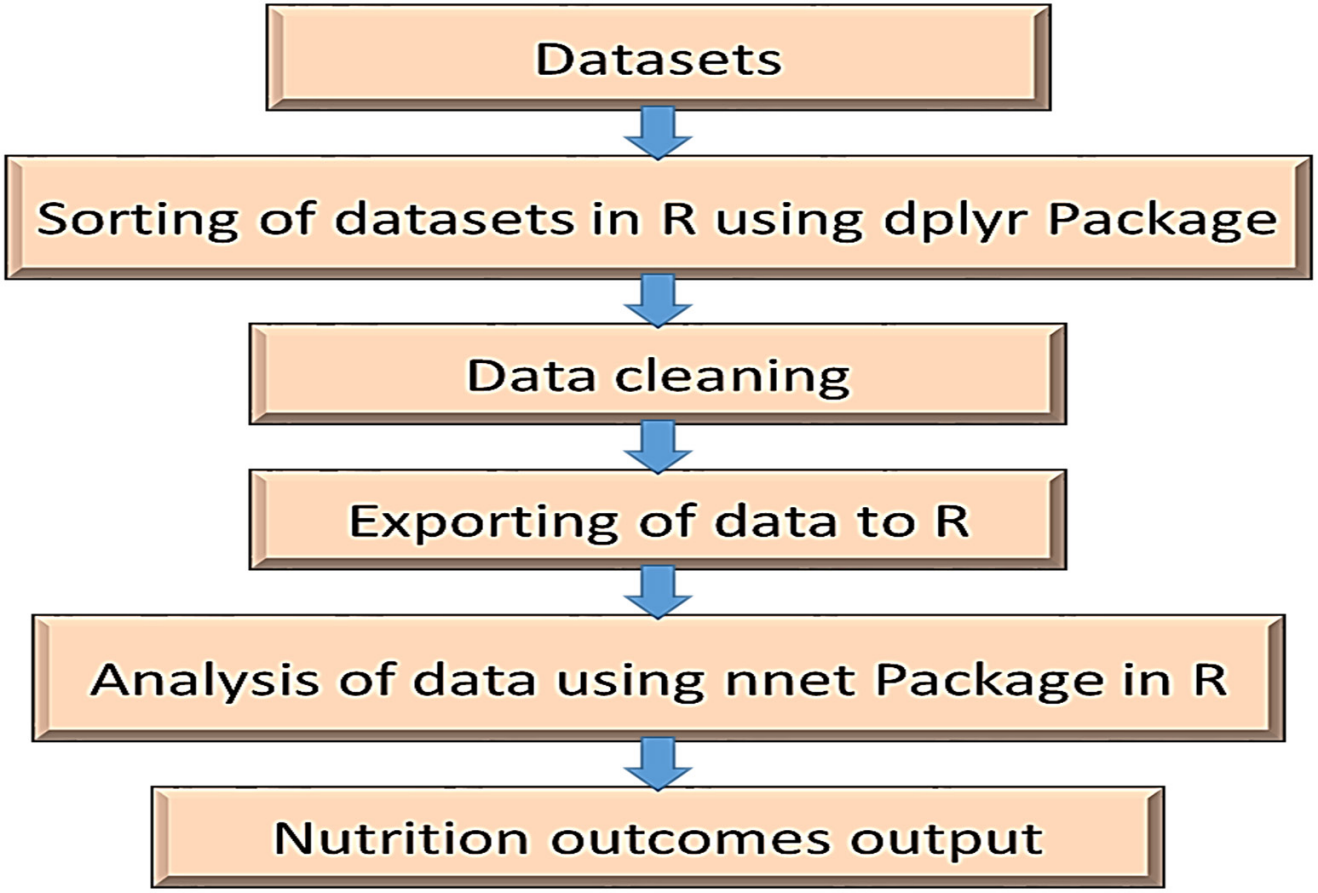
Flow chart of analyses in R‐Studio. Source: Authors.

Figure 2 presents the flow chart of the data analyses in R‐Studio software. It depicts the details of the process through which the research can be reproduced or adapted, especially on similar datasets or allied.

## 4 RESULTS AND DISCUSSION

4

### 4.1 Descriptive statistics of food price

**FIGURE 3:**
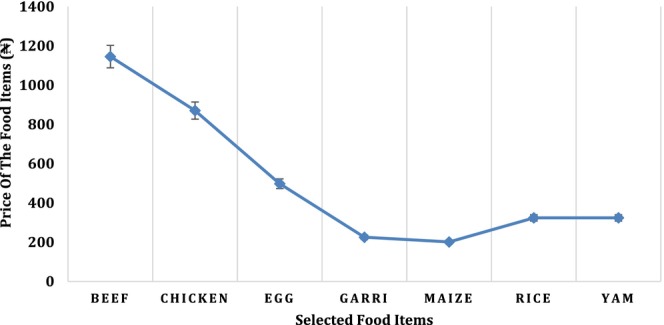
Price trends of the selected food items for 2018. Source: Computed from Nigeria Bureau of Statistics data (2018).

Figure 3 shows a broad picture of the prices of selected food items in Nigeria. The prices are average regional market prices over a 12‐month period for the year 2018. Beef is the most expensive of all, with an average price of ₦1146.07 per kg. This is closely followed by the average price of chicken ₦876.24 per kg. The rise in the prices of food items compared to previous years (≤ 2017) is largely triggered by the instability of the exchange rate of naira, insecurity, and high cost of transportation of products within and across states in the country (NBS, 2018). The least expensive is maize, costing about ₦200.84 per kg as presented in Figure 3.

### 4.2 Description of the outcome variable (BMI) for women

**FIGURE 4:**
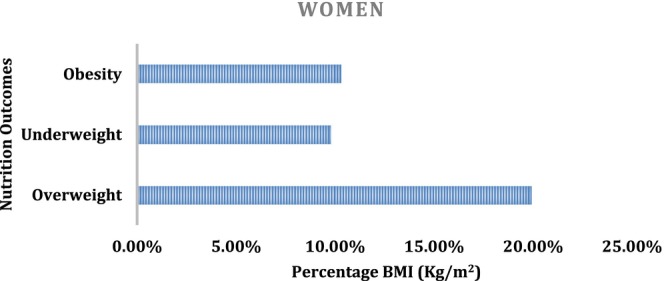
Percentage distribution of nutrition outcomes among women in Nigeria. Source: Computed from NHDS (2018).

Figure 4 shows the prevalence of the different body weight categories among the women studied. The percentage distribution of nutrition outcomes is shown in Figure 4. Overweight was 19.9%, whereas underweight and obesity had 9.8% and 10.3%, respectively. This is contrary to the report that said the prevalence of obesity among women in Eswatini is higher at 23% (Neupane et al., 2015). The percentage sum total of nutrition outcomes (overweight, underweight, and obesity) is 40% which implies that the remaining 60% of the respondents had normal body weight. The BMI ratios were not consistent with the studies of adolescent women in Afghan refugees with 16.7% of participants having a normal weight and 8.6% were overweight (Fatima et al., 2023). This might be a result of their higher level of education, physical activities, and less consumption of more processed food (Alamu et al., 2018; Onyeaka et al., 2023; Ostojic et al., 2021).

### 4.3 Multinomial logistic regression results

A multinomial logistic regression model was developed for marginal effects (probabilities) and their standard errors (Table 3) to establish the relationship between nutrition outcomes and food prices of energy‐dense and nutrient‐dense food items as well as their significant factors.

**TABLE 3:** BMI multinomial logit regression for energy dense among Nigerian women.

Variable	Dependent variable		
	Obesity (1)	Overweight (2)	Underweight (3)
“Place of residence” (Urban ref.)	−1.468*** (0.186)	−1.013*** (0.195)	0.298 (0.225)
“Wealth status” (poor ref.)	−0.034 (0.088)	−0.049 (0.069)	−0.141 (0.095)
Age	0.005 (0.024)	−0.019 (0.019)	−0.002 (0.024)
Region (South‐South ref.) North‐Central	−0.596*** (0.164)	−0.289** (0.133)	−0.147 (0.167)
Region North‐East	−0.581*** (0.175)	0.019 (0.131)	−0.251 (0.179)
Region North‐West	0.043 (0.163)	0.051 (0.129)	−0.229 (0.187)
Region South‐East	0.01 (0.161)	0.075 (0.128)	−0.087 (0.176)
Region South‐West	−0.242 (0.17)	−0.069 (0.134)	0.012 (0.162)
Education (tertiary education dummy) “No formal education”	−0.16 (0.162)	−0.036 (0.125)	0.106 (0.159)
“Primary education”	−0.061 (0.162)	−0.099 (0.129)	0.104 (0.158)
“secondary education”	−0.121 (0.148)	0.06 (0.115)	0.075 (0.141)
“Energy dense price”	0.002*** (0.001)	0.003*** (0.001)	−0.005*** (0.002)
Constant	−0.485** (0.199)	−0.916*** (0.352)	−0.490** (0.219)
Akaike Inf. Crit.	16,617.62	16,617.62	16,617.62

*Note*: *,**,***p < .01. *Means significant at 10% (0.1). **Means significant at 5% (0.05). ***Means significant at 1%; (0.01); Number of observations = 4907; Pseudo‐*R* ^2^ = 0.05912; log‐likelihood = −2930.9. Bracketed Figures are the standard errors. Source: Authors.

Nutrition outcomes reference or base group is normal weight, place of residence reference is urban area, wealth status reference is poor, and geopolitical zones reference is South‐South, Nigeria, because food items are very expensive in the zones.

As shown in Table 3, the coefficient of energy‐dense price has a positive relationship with obesity and overweight and has an inverse relationship with underweight. This means that a rise in cost of any energy‐dense food price by 1 unit will reduce the probability of being undernourished relative to normal weight and is expected to decrease by 0.5% and it is statistically significant at a 1% level. Similarly, the coefficient of energy‐dense food price has a positive relationship with obesity and overweight and it is statistically significant at 1% as seen in Table 3, this means that increase in energy‐dense food price by 1 unit will reduce the probability of being obese and overweight by 0.2% and 0.3%, respectively, relative to normal weight as evidenced in Table 3. This conforms to the study (Ajetomobi et al., 2019; Neupane et al., 2015) that a 1% increase in the ratio of oil price and maize price (energy dense) will lead to about 0.96% decline in BMI for the respondent. Table 3 shows that place of residence, with urban area as reference category, the marginal effect for the rural area of 0.29 shows that the expected probability of being undernourished is higher for women living in rural areas compared to urban areas by 0.29 when the other independent variables are held constant at their means. This trend is suggested to have been influenced by several factors such as the shift in progress in alleviating programs for malnutrition in rural areas and also the pronounced poverty in the rural area (Li et al., 2021; Mekonnen et al., 2021; Park et al., 2023). Also, it is suggested to be a result of the rise in the cost of food; for example, in the year 2017, the price of food in Nigeria was double the price in 2020 due to increase in the cost of production and poor harvest of the crops. It could also be a result of recessions in the country and globally.

There is a negative relationship between obesity and overweight with the place of residence (rural area) compared to urban area as shown in Table 3. This implies that the probability of being obese and overweight is low among women living in rural areas compared to the urban area, this follows the apriori expectation. This might be because rural residents had higher energy expenditure in their daily work, especially in agricultural and domestic activities like fuelwood and water collection (Saaka et al., 2017). Also, it might be due to lower incomes in rural areas restricting food consumption, but having access to fresh fruits at different seasons (Hoque et al., 2021; Perrin et al., 2018). It might also be as a result of less access to and use of public transport which makes them walk and this helps them in burning down some excess fats than the sedentary lifestyles in the urban areas since the World Health Organization recommends exercises in the form of walking for healthy living (Owade et al., 2020; Yankah et al., 2020). The findings are in conformity with those reported by Neupane et al. (2015) that women living in urban areas had higher likelihood of being overweight and obese. However, the coefficient of age has a positive relationship with obesity, this suggests that age is more likely to be associated with obesity. This is congruent with the findings that there is a positive relationship between age and obesity (Ajetomobi et al., 2019; Asfaw, 2006; Chou et al., 2004). The coefficients of the region for North‐Central for BMI were − 0.927 for obesity, −0.613 for overweight, and 0.244 for underweight. The coefficients of region for North‐Central variable were negative for obesity and overweight but positive for underweight and statistically significant at 1% probability level. Comparatively, the marginal effect for North‐Central region shows that expected probability of being undernourished is higher among the women living in the region compared to South–South while other independent variables are held constant at their means. This may be attributed to issues of poverty which is more pronounced in the northern part of Nigeria.

Table 4 presents the results of BMI regression analysis for nutrient‐dense food price and personal environment characteristics among women.

**TABLE 4:** BMI multinomial logit regression for nutrient dense among Nigerian women.

Variable	Dependent variable		
	Obesity (1)	Overweight (2)	Underweight (3)
“Place of residence” (urban ref.)	−0.829*** (0.057)	−0.541*** (0.040)	0.194*** (0.058)
Wealth Index (poor ref.)	0.075 (0.086)	0.011 (0.066)	−0.117 (0.091)
Age	0.017 (0.025)	−0.009 (0.018)	−0.004 (0.024)
Region (South‐South ref.) Region North‐Central	−0.927*** (0.096)	−0.613*** (0.075)	0.244*** (0.093)
Region North‐East	−0.580*** (0.098)	−0.102 (0.067)	−0.033 (0.089)
Region North‐West	−0.061 (0.077)	−0.002 (0.060)	−0.155 (0.097)
Region South‐East	0.055 (0.109)	0.148* (0.082)	−0.075 (0.115)
Region South‐West	−0.085 (0.110)	0.004 (0.080)	0.167* (0.095)
Education (tertiary education ref.) “No formal education”	−0.114* (0.064)	−0.004 (0.050)	0.097 (0.066)
“Primary education”	−0.087 (0.069)	−0.132** (0.054)	0.145** (0.068)
“Secondary education”	−0.134* (0.069)	0.043 (0.053)	0.090 (0.066)
“Nutrient dense food price”	0.003*** (0.002)	0.004*** (0.002)	−0.0018*** (0.001)
Constant	−5.776*** (0.012)	−4.469*** (0.008)	0.196*** (0.013)
Akaike Inf. Crit.	16,544.350	16,544.350	16,544.350

*Note*: *,**,***p < .01. *Means significant at 10% (0.1); **Means significant at 5% (0.05); ***Means significant at 1%; (0.01); Number of observations = 4907; Pseudo‐*R* ^2^ = 0.04912; log‐likelihood = −2730.9. Bracketed figures are the standard errors. Source: Authors.

Table 4 shows that the coefficients of nutrient‐dense food prices are positively significant at 1% for the nutrition outcomes (obesity and overweight). This means that a rise in cost of any nutrient‐dense food price by 1 unit will reduce the probability of being obese and overweight relative to normal weight and is expected to decrease by 0.3% and 0.4%, respectively. However, it has inverse relationship with undernutrition by 0.1%. This implies that nutrient‐dense food price is more associated with obesity and overweight compared to undernutrition. This might be associated with food production modernization and rising income levels which allow them to consume more meat, capable of increasing weight gain due to its high‐fat content. This is similar to the findings that, fats are not absorbed for energy provision as stated; fats absorption happens in different manner compared to carbohydrates (Grantham et al., 2014). Furthermore, the place of residence has a negative statistical and significant effect on the probability of being obese or overweight relative to normal weight. It might be a result of access to and intake of fresh varieties of fruits and vegetables among women living in rural areas (Adeleke & Babalola, 2020). Also, nonavailability of sedentary lifestyle among rural women who were involved in different physical activities on their farms. This is consistent with the findings of Ajetomobi et al. (2019), which revealed that there is a statistically significant relationship among wealth status, age, and probability of being overweight, obese, and underweight among women. Evidently, from Table 4, the level of education is one of the determining factors for nutrition outcomes. It means that the low level of education increases the chance of being undernourished; the slight variations could be attributed to anthropogenic activities and the type of food intake of the respondents.

## 5 CONCLUSIONS

This study has established that the prevalence of overweight (19.9%) and obesity (10.3%) was the major problems of nutrition outcomes among Nigerian women. The empirical findings from this study provide satisfactory evidence that food prices affect nutrition outcomes. Using a multinomial logit regression model, the study revealed that increases in food prices are associated with nutrition outcomes (underweight, overweight, and obesity) among women, which is significant at p‐value of 0.01. The reduced‐form price‐effect models revealed that the BMI of women is affected by food prices and environmental characteristics. In contrast to apriori expectation, BMI is positively related to the price of nutrient‐dense food.

## 6 RECOMMENDATIONS

The findings from this study showed that nutrition outcomes are sensitive to food prices. The current study recommends that government should let people have free access to formal education, especially in rural areas. Organizing workshops or seminars for households about health and nutrition promises to drastically reduce the prevalence of nutrition outcomes. Also, a policy approach that seeks to suppress food price increment will have long‐term consequences for poverty reduction by constraining the potentially positive effect of food prices on women's undernutrition. In the event of poverty existence, the populace may divert to plant protein like soybeans and beans which can be used to complement energy‐dense food in the household since animal protein is very expensive.

The promotion of affordable nutrient‐rich foods, particularly vegetables, dairy products, and protein‐rich foods, must be intensified without compromising environmental sustainability in the rural and urban areas. Therefore, a backyard garden is encouraged among households in the urban area since it is a good way for low‐income families to save money and gain quick access to fruits and vegetables. It would provide an avenue through which the populace can undertake cultivations peculiar to their health growths. The Nigerian government and other nations with prevalence of nutrition outcomes (underweight, overweight, and obesity) should consider introducing a pricing policy that encourages the production and consumption of food items with nutritive value to curb the prevalence. Additionally, nutrition education should be promoted among women of diverse household status both in rural and urban areas.

## AUTHOR CONTRIBUTIONS

**Deborah Tosin Fajobi:** Conceptualization (lead); data curation (lead); writing – review and editing (equal). **Joshua Olusegun Ajetomobi:** Project administration (equal); resources (equal); supervision (lead). **Mufutau Oyedapo Raufu:** Project administration (equal); supervision (lead); writing – review and editing (supporting). **Moses Oluwatobi Fajobi:** Investigation (equal); writing – original draft (lead); writing – review and editing (equal). **Prabhu Paramasivam:** Project administration (supporting); supervision (lead); writing – original draft (supporting); writing – review and editing (lead).

## FUNDING INFORMATION

This research did not receive any specific grant from funding agencies in the public, commercial, or not‐for‐profit sectors.

## CONFLICT OF INTEREST STATEMENT

The authors declare no conflict of interest.

## Data Availability

The data used to support the findings of this study are included in the articles. Should further data or information be required, these are available upon reasonable request from the corresponding author.
